# Breast malignancy in female-to-male transsexuals: systematic review, case report, and recommendations for screening

**DOI:** 10.1016/j.breast.2020.06.008

**Published:** 2020-07-02

**Authors:** Anne C. Fledderus, H. Antoine Gout, Aernout C. Ogilvie, Dorothea K.G. van Loenen

**Affiliations:** a Amsterdam UMC, University of Amsterdam, Department of Plastic, Reconstructive and Hand Surgery, Meibergdreef 9, Amsterdam, the Netherlands; b Department of Internal Medicine, Medical Oncology, Onze Lieve Vrouwe Hospital, Jan Tooropstraat 164, Amsterdam, the Netherlands; c Medisch Centrum Bloemendaal, Zomerzorgerlaan 50, Bloemendaal, the Netherlands

**Keywords:** Transsexual, Transgender, Breast malignancy, Breast cancer, Breast neoplasm, Hormone therapy, Testosterone, FtM transsexuals, female-to-male transsexuals, DCIS, ductal carcinoma in situ, HER2, Human Epidermal growth factor Receptor 2

## Abstract

**Background:**

Female-to-male (FtM) transsexuals may use testosterone therapy for masculinization, which potentially influences the risk of breast cancer development. Guided by our case report, we aimed to investigate the evidence regarding the risk of testosterone therapy on breast malignancy in female-to-male transsexuals and evaluate breast cancer screening in this subgroup.

**Methods:**

We conducted a systematic literature search according to the PRISMA checklist in June 2020 in PubMed/MEDLINE and Ovid/EMBASE. Reference lists of included articles were screened to find additional articles that met the inclusion criteria. All cohort studies and case reports evaluating breast cancer in FtM transsexuals after testosterone therapy were included.

**Results:**

We found 23 cases of FtM transsexuals who developed breast cancer after testosterone therapy, including our own case. Moreover, we evaluated ten retrospective cohort studies investigating breast malignancy in the transsexual population. The cohort studies showed no elevated risk in FtM transsexuals compared to natal women. Including our own case, nine cases were described in which breast malignancy was incidentally found during routine histological examination after mastectomy. High-level evidence for a correlation between testosterone therapy and breast malignancy is missing.

**Conclusion:**

Few cases are described of FtM transsexuals with breast malignancy. However, cases such as these make physicians aware of the possibility of breast cancer in FtM transsexuals. Radiological screening of FtM transsexuals for breast cancer prior to mastectomy and histological screening of the mammalian tissue after mastectomy should be considered; physicians should decide together with every individual FtM transsexual if screening is necessary.

## Highlights

• Breast cancer malignancy after testosterone therapy in female to male (FtM) transsexuals is very rare, including our own case, we found 23 of such cases reported in literature.
• Based on current evidence, it is not possible to conclude associations between FtM transsexuals using testosterone therapy and breast cancer.
• Radiological screening FtM transsexuals for breast cancer prior to mastectomy and histological screening of the mammalian tissue after mastectomy should be considered; physician should decide together with every individual FtM transsexual if screening is necessary.

## Background

Breast cancer is the most common malignancy and the most frequent cause of death from cancer in natal women [1]. The risk of developing breast cancer in female-to-male (FtM) transsexuals is unknown [2]. FtM transsexuals could be at extra risk due to the testosterone therapy they may receive for masculinization [3], [4]. The associations between testosterone and breast cancer are debated in the literature. Proliferative effects of testosterone on breast cancer are described, but antiproliferative effects are suggested as well [5]. It is unclear if FtM transsexuals with a history of testosterone use have a higher or lower risk of developing breast cancer [2]. We aimed to provide an insight into the reported number of FtM transsexuals developing breast cancer after testosterone therapy by systematically reviewing the literature and evaluating the evidence regarding associations between testosterone therapy and breast cancer. In addition, we report a case of an FtM transsexual with a history of testosterone use, in whom a neoplasm was found in the breast tissue after mastectomy.

## Methods

We conducted a systematic review, reported according to the PRISMA checklist [6]. A search was performed in June 2020 in PubMed/MEDLINE and Ovid/EMBASE (Appendix 1). Furthermore, reference lists of included articles were screened to find additional articles that met the inclusion criteria. All articles evaluating breast cancer in FtM transsexuals after testosterone therapy were deemed eligible, including case reports, case series, cohort studies, and case-controlled studies. Two reviewers (ACF, HAG) independently screened titles and abstracts. Full texts of potentially eligible studies were critically reviewed to assess eligibility. Disagreement on inclusion was resolved through discussion.

Data extraction was performed in duplicate by two independent reviewers (ACF, HAG). The following data was extracted from the FtM transsexual cases with testosterone use developing breast malignancy: age of diagnosis of breast malignancy, whether this diagnosis was prior to or after subcutaneous mastectomy, family history of breast malignancy, testosterone use in years, tumor type, BRCA gene status, hormone receptor status. The Joanna Briggs Institute’s critical appraisal tools were used to perform a quality assessment of the included studies [7]. We evaluated every case individually with the risk of bias tool for case reports. The cohort study risk of bias tool was used to evaluate the evidence regarding the relation between testosterone use and breast cancer development investigated by the included cohort studies. We did not exclude articles based on their quality, as we aimed to give an overview of available evidence. Data synthesis was performed by using descriptive statistics.

### Case report

We reported a case of an FtM transsexual with breast neoplasm after testosterone use. The casereport was reported according to the CARE checklist for case reports [8].

## Case report

Our case concerns a 50-year-old Caucasian FtM transsexual and ex-bodybuilder, who received 3 years of hormone therapy. He used one year of testosterone therapy for induction of masculinization, and two years of anabolic-androgenic steroids for muscle enlargement. At the time of presentation, there were no clinical or psychosocial health issues, there were no abnormalities found during the physical examination, he had no overweight, and used no medications other than testosterone.

The patient’s mother was diagnosed with breast cancer at the age of 50. No other first or second-degree family members with a history of breast or ovarian cancer were recalled. BRCA gene testing was not performed.

Because of the planned mastectomy, the patient refrained from the national population screening program for breast cancer and did not undergo mammography before mastectomy.

**Fig. 1:**
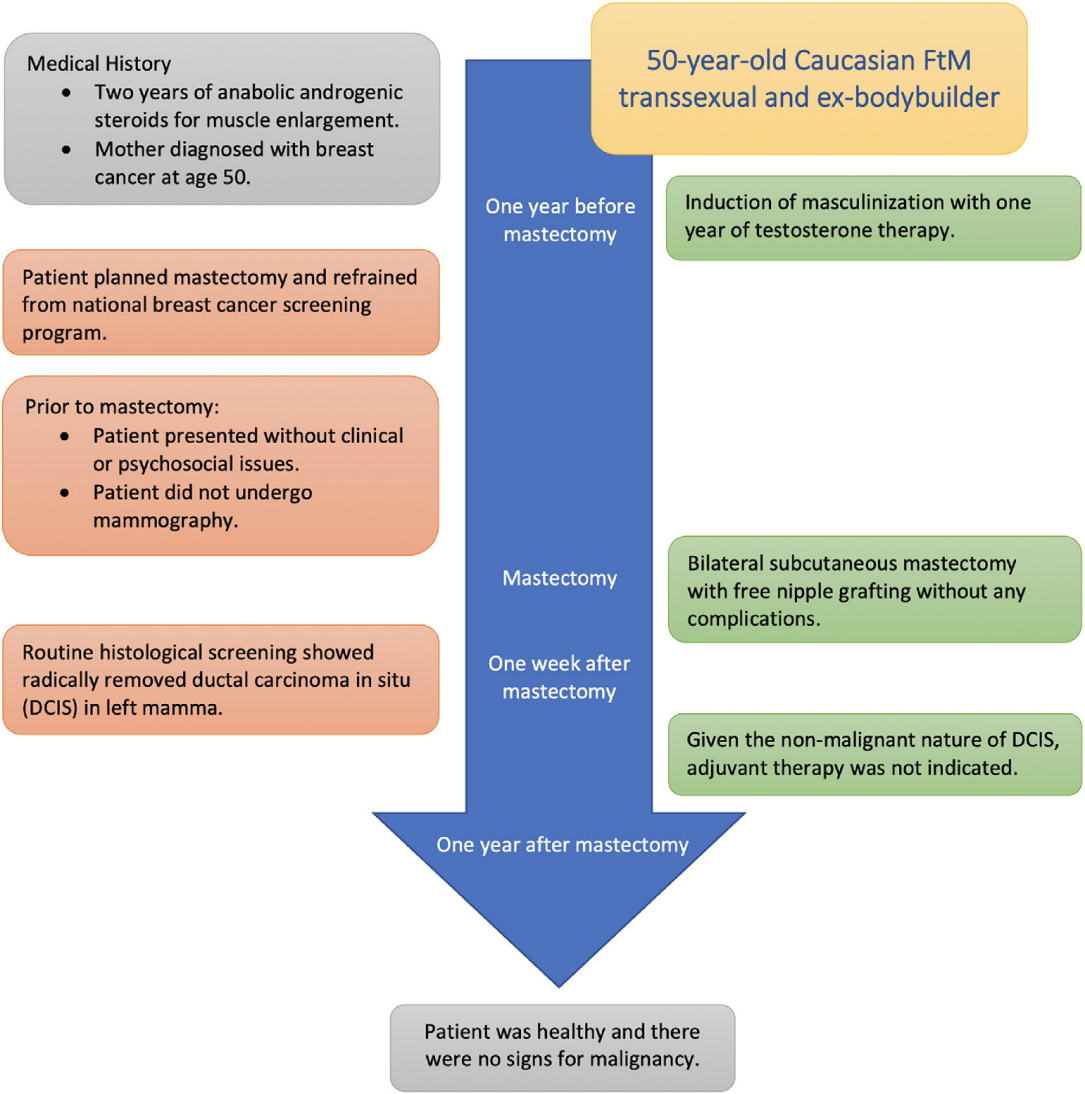
Timeline of the case report.

A bilateral subcutaneous mastectomy with free nipple grafting was performed in the Slotervaart Hospital in Amsterdam, The Netherlands. The operation was without any complications. Routine histological examination with hematoxylin and eosin-staining of this breast tissue showed ductal carcinoma in situ (DCIS) in the left mamma. This premalignant tumor proved to have been radically resected by the mastectomy for gender reassignment. As there was no evidence of invasive cancer, no additional oncological surgery nor local radiotherapy were necessary [9]. Because of the non-invasive nature of DCIS, no adjuvant systemic therapies with endocrine or chemotherapy was indicated [9]. Hence, the hormone receptor status of the tumor was not assessed. Moreover, any future risk of breast cancer had been minimalized by the subcutaneous mastectomy [10]. Our patient continued his testosterone therapy. One year after the subcutaneous mastectomy, he was healthy and there were no signs of malignancy. Fig. 1 describes the timeline of our subject. Our patient reported a negative impact of these events on his psychological well-being. He explained that breast malignancy is a disease commonly affecting women. The management and checkups for the breast neoplasm did therefore not feel to be in line with his male gender identity. He gave verbal informed consent for this study.

## Results

**Fig. 2:**
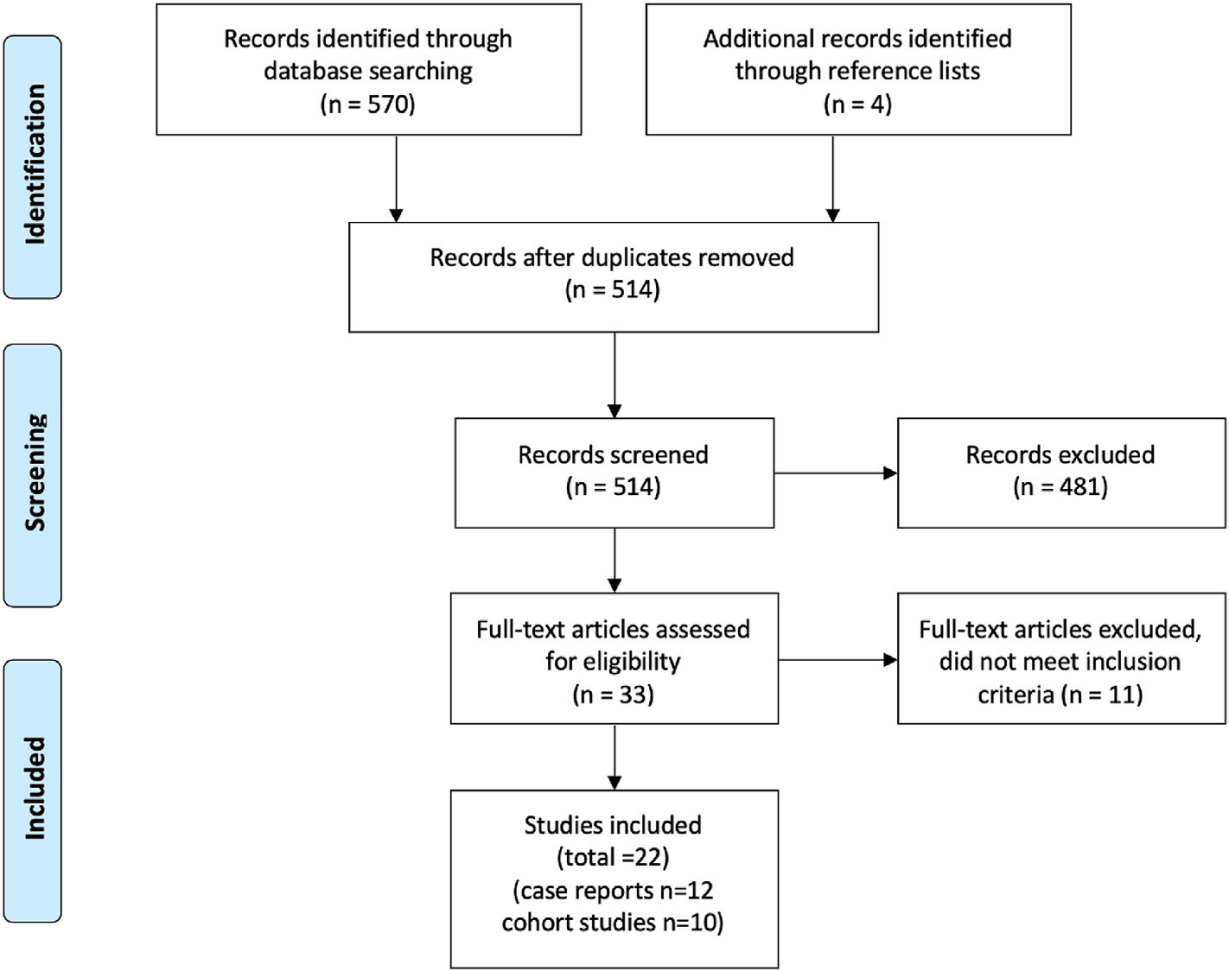
Flow diagram of the article selection.

We screened 510 articles and 22 studies (12 case reports, 10 cohort studies) were included in our review. Fig. 2 shows the flow diagram of the study inclusion process. We found 23 cases describing breast cancer in FtM transsexuals with a documented history of testosterone use, including our own case. These cases were described in case reports (n = 12) or cohort studies (n = 4). Details of these cases are listed in Table 1. The age of the subjects, when age was reported (n = 19), ranged from 27 to 77 years (42 yr mean; 41 yr median). A total of sixteen patients was younger than 50 and seven patients were 50 or older. The duration of testosterone use ranged from four months to 25 years (7 yr mean; 4 yr median). In nine cases a positive family history of breast cancer was reported, and BRCA gene-testing was negative in five of these cases. Further information on BRCA status was lacking in all other subjects. Fourteen of the tumors were described to be of ductal origin. Estrogen and progesterone receptor status were reported in twenty and nineteen cases respectively. Estrogen was positive in seventeen, negative in three, and progesterone was positive in thirteen and negative in six. The HER2 receptor was reported in thirteen cases. It was positive in seven and negative in six. The androgen receptor was tested in six subjects and positive in five and negative in one. One triple-negative breast cancer was reported.

**Table 1:** Cases of Female-to-Male transsexuals developing breast cancer after testosterone therapy.

Author	Study Design	Age (in years) at diagnosis	Mastectomy prior to diagnosis	Family history	Testosterone use in years	Tumor type	BRCA status	Receptor status
Barghouthi et al. (2018) [22]	Case report	28	No	Paternal great grandmother with breast cancer, maternal great grandmother with ovarian cancer.	1	Invasive ductal carcinoma grade 3	Negative	ER-, PR-, AR-, HER2+
Brown & Jones (2015) [16]	Retrospective cohort study	77	Unknown	Unknown	11	Unknown	Unknown	ER+, PR-
Burcombe et al. (2003) [23]	Case report	33	Yes (10 years after mastectomy)	Negative	13	Ductal carcinoma	Unknown	ER+, PR+
Chotai et al. (2019) [24]	Case report	58	Yes (20 years after mastectomy)	Positive	10	Invasive ductal carcinoma grade 3	Unknown	ER+, PR+, HER2+
De Blok et al. (2019) Describes four cases (n = 4) [20]	Retrospective cohort study	30-50 (n = 2), >50 (n = 2)	Yes (n = 3) (“several years after mastectomy”) and Yes, incidental finding (n = 1)	Unknown	Median 15; range 2-17	Ductal origin (n = 3)	Unknown	ER+(n = 2), PR+ (n = 2), HER2+ (n = 1), AR+ (n = 1)
Eismann et al. (2019) and Baker et al. (2019) [19, 25]	Case report and retrospective cohort study	29	Yes, incidental finding	Positive	4	High-grade DCIS	Negative	ER+
Fundytus et al. (2019) [26]	Case report	48	Yes, incidental finding	Positive	19	Invasive ductal carcinoma	Unknown	ER+, PR+, AR+, HER2-
Gooren et al. (2013) [17]	Retrospective cohort study	27	Yes, incidental finding	Unknown	3	Tubular adenocarcinoma	Unknown	ER +, PR+
Gooren et al. (2015) [27]	Case report	41	Yes, incidental finding	Unknown	1	Tubular adenocarcinoma	Unknown	ER+, PR+, HER2-
Gooren et al. (2015) [27]	Case report	48	Yes	Unknown	9	Infiltrative ductal carcinoma	Unknown	ER-, PR-, HER2 -
Katayama et al. (2016) [28]	Case report	41	Yes (12 years after mastectomy)	Negative	15	Invasive ductal carcinoma	Unknown	ER+, PR+, HER2–
Light et al. (2020) [29]	Case report	44	No∗	Unknown, due to adoption	4 months	Invasive ductal carcinoma grade 2	Unknown	ER+, PR+, AR+, HER2-
Nikolic et al. (2012) [30]	Case report	42	Yes (1 year after mastectomy)	Negative	2.5	Invasive ductal carcinoma	Not Tested	ER-, PR-, AR+, HER2+
Shao et al. (2011) [31]	Case report	27	No∗	Positive	6	Invasive ductal carcinoma grade 3	Negative	ER+, PR+, HER2+
Shao et al. (2011) [31]	Case report	53	No∗	Positive	5	Invasive ductal carcinoma grade 2	Negative	ER+, PR, HER2+
Tanini et al. (2019) [32]	Case report	33	Yes, incidental finding	Positive	2.5	DCIS grade 3	Unknown	ER+, PR+, AR+
Tanini et al. (2019) [32]	Case report	36	No	Positive	3	Poorly differentiated invasive carcinoma of no special type	Negative	ER+, PR+, HER2+, AR+ (60%)
Treskova et al. (2018) [33]	Case report	58	Yes, incidental finding	Unknown	25	Invasive ductal carcinoma	Unknown	ER+, PR-, HER2-
Van Renterghem et al. (2018) [18]	Retrospective cohort study	31	Yes, incidental finding	Negative	1.3	Moderately differentiated invasive carcinoma	Unknown	ER+, PR+, HER2-
Fledderus et al. (2020) (current article)	Case report	50	Yes, incidental finding	Positive	3	DCIS	Not tested	Not tested

No∗: suspicion for malignancy, diagnosis confirmed by histological examination after mastectomy; Yes, incidental finding: by histological analysis after mastectomy; ER: estrogen receptor; PR: progesterone receptor; HER2: Human Epidermal growth factor Receptor; AR: androgen receptor; -: negative; +: positive; DCIS: ductal carcinoma in situ.

We found ten retrospective cohort studies evaluating breast cancer in FtM transsexuals with a history of testosterone therapy. In five of these cohort studies, no cases of breast cancer were found [[11], [12], [13], [14], [15]]. These cohort studies, however, were relatively small: respectively 293, 112, 133, and 56 FtM transsexuals with hormone therapy [[11], [12], [13], [14], [15]]. One of these studies only found a case of breast malignancy in the group of FtM transsexuals not using testosterone (n = 130), concluding that there is no elevated risk for individuals using testosterone compared to those not using testosterone [15]. A study with 1579 American veteran FtM transsexuals found seven FtM transsexuals with breast cancer, whereas only one was documented to use testosterone prior to diagnosis [16]. Three cohort studies including 795, 96, and 283 FtM transsexuals receiving testosterone all found one subject with breast malignancy or premalignancy in their cohort [17], [18], [19]. A recent Dutch study evaluated the incidence of breast cancer in the FtM transsexual population in their clinical center [20]. In their population of 1229 FtM transsexuals, four cases of invasive breast cancer were diagnosed [20]. According to the findings of this study, this FtM transsexual population has a lower risk of developing breast cancer compared to Dutch natal women, but a higher risk compared to Dutch natal men [20]. Note that the studies by Goren et al. (2013 & 2015) [17,21] were performed in the same clinical center as the recent Dutch study (2019) [20]. Two of the four cases described by this Dutch study might have been the same as those in Goren et al. (2013 & 2015) [17,21].

### Quality assessment

**Fig. 3:**
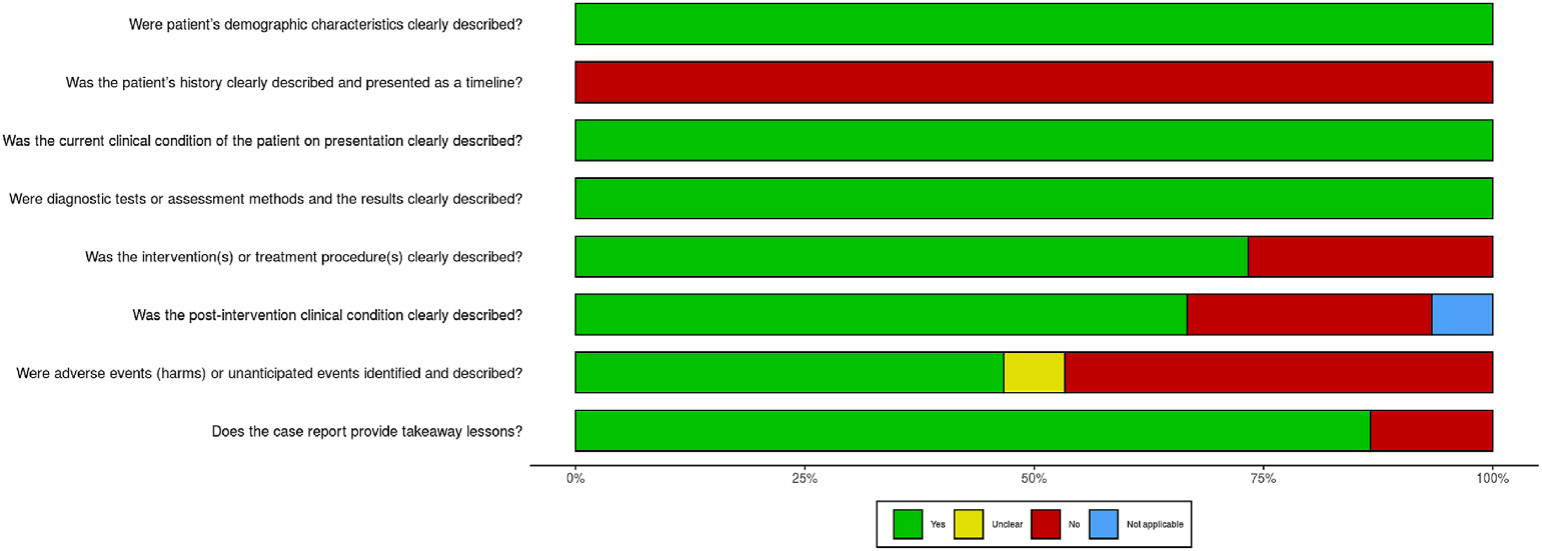
Risk of bias of the case reports.

**Fig. 4:**
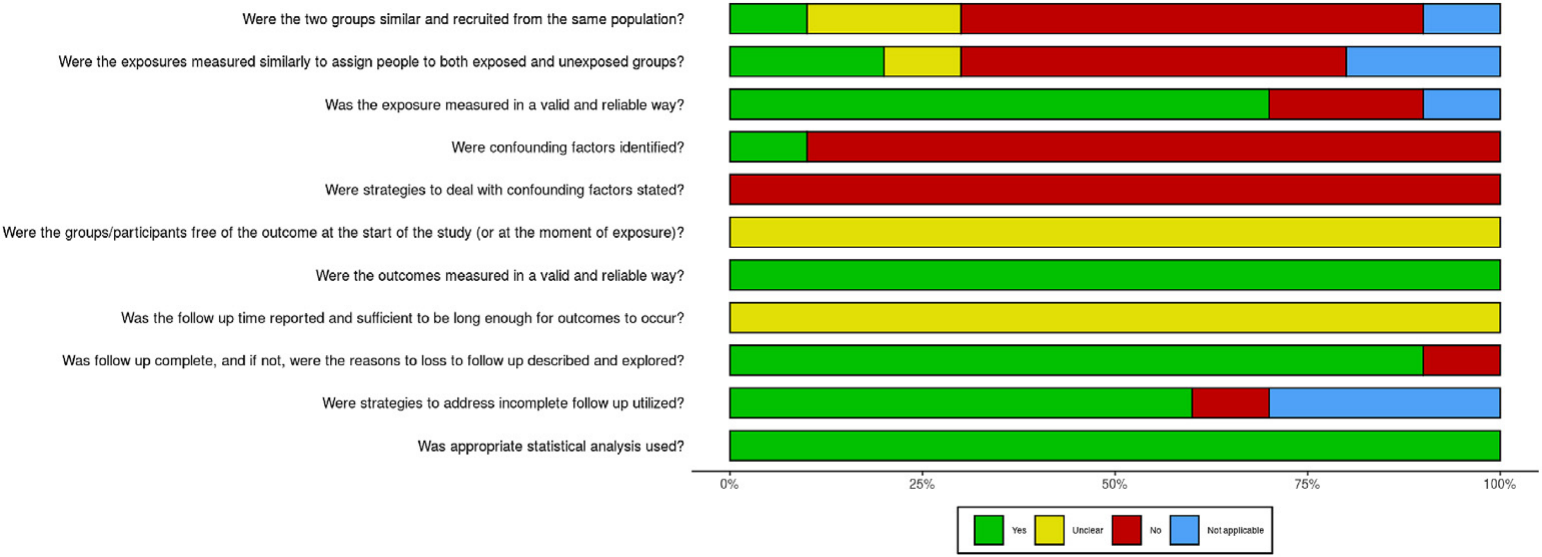
Risk of bias of the cohort studies.

Fig. 3 (case reports) and Fig. 4 (cohort studies) show the results of the quality assessment by means of the two JBI tools. In Appendix 2, the evaluations of every individual case report and cohort study are shown. Three cohort studies formed a control group [15,18,19] and six studies collected data of cancer incidence in the general population as a control [11,13,14,16,17,20]. One study did not make use of a control group [12]. Six studies stratified patients based on age and sex [11,13,14,16,17,20]. Several confounding factors were reported, including a history of mastectomy [12, 13, 15, 18, 19], family history [19], bodyweight/BMI [18, 19, 34], smoking status [14, 19, 34], and alcohol use [19], but none corrected for these in the analyses. The JBI and CARE checklist recommend presenting the patient’s historical and current information as a visual timeline, but none of the studies did accordingly [8]. Eight case reports did not clearly describe the treatment outcomes of the breast cancer; no post-intervention clinical condition was reported [22, 24, 32], no harmful events as a result of interventions were described [22,24,25,30,32,33], or the patient had not completed treatment yet [26].

## Discussion

Data on breast cancer in FtM transsexuals with a history of testosterone use is limited to the cases described in Table 1. The incidence of breast cancer cases described in the FtM transsexuals cohort studies is shown to be significantly lower than the breast cancer incidence in natal women [16], [17], [20]. Due to the low level of evidence (case reports) and high risk of bias of the included cohort studies, it is not possible to conclude a correlation between hormone therapy and the development of breast cancer.

All cohort studies and case reports did not report screening for cancer in advance of testosterone therapy, which complicates the evaluation of its potential causal relationship with cancer. Whether follow-up was sufficiently long enough (range: < 1 yr - >30 yr) could not be determined for any study, since the course of cancer development in these patients is still unclear. Moreover, younger patients may demand a longer follow-up, since their baseline cancer risk is lower than those of older patients. Confounders were poorly described and none was appropriately corrected for in the analysis. The most important confounder for investigating the effect of testosterone on breast malignancy is a history of mastectomy. The lower incidence of breast cancer found in the studies can be attributed to the performed mastectomies. Mastectomy is often the first and only step of gender reassignment surgery of FtM transsexuals [35]. After mastectomy with nipple reimplantation, the risk of breast cancer in an FtM is reduced by nearly 90% [10].

Nonetheless, breast tissue is often not radically removed when the indication for a subcutaneous mastectomy is sex reassignment for FtM transsexuals [36]. This may explain the seven rare cases describing breast cancer development after mastectomy [20,23,24,28,30]. The 23 subjects with breast cancer were relatively young (mean age 42 years). A large number of older FtM transsexuals may have undergone a mastectomy, which decreases the risk of malignancy in older individuals [10,24]. The majority of subjects had an invasive tubular carcinoma, which is in line with the general population; an estimated 76% of the invasive tumors are of tubular origin [37].

In the literature, two theories suggest an association between breast cancer development and testosterone therapy. The first theory suggests an increased risk of breast cancer because testosterone is aromatized into estradiol, which in turn is associated with breast tumor growth [28,38]. Associations between elevated endogenous testosterone levels and breast cancer development have been shown by several studies [[39], [40], [41]]. It has been suggested that higher blood plasma levels of androgens can specifically increase the risk of hormone-receptor-positive breast cancer [[42], [43], [44], [45]]. Furthermore, one study showed that androgens could decrease the risk of developing receptor-negative breast cancer [46]. This could explain the relatively high presence of hormone-positive breast cancer found in our subjects and relatively low number of individuals with hormone negative breast cancer. HER2-positive tumors were reported in 37% (7/19) of the invasive carcinomas. In the general female population, HER2-positive tumors are estimated to be 15% of invasive carcinomas [47]. However, the individuals included in our study were relatively young and younger individuals with breast cancer tend to have a higher incidence of HER2-positive breast cancer [48].

In contrast to the first theory, the second suggests that testosterone reduces breast cancer risk [49, 50]. One study showed a lower incidence of breast cancer in women using testosterone therapy [50]. A preclinical study supports this theory, showing both a proliferative effect of androgens and an antiproliferative effect of androgens mediated by the androgen receptor [5]. Moreover, testosterone use reduces breast tissue density [51], and dense breast tissue has been shown to be a risk factor for breast malignancy [52]. Another risk factor for breast malignancy is a high number of menstruation cycles and high lifetime menstruation activity [53]. Testosterone could have an antiproliferative effect by suppressing the menstruation cycles in FtM transsexuals [13]. The ‘Standards of Care for the Health of Transsexual, Transgender, and Gender-Nonconforming People’ state that there is no increased risk of breast cancer for individuals treated with testosterone [4].

Breast cancer screening prior to mastectomy was not performed in our case. Luckily, the neoplasm was completely removed after mastectomy. However, when breast tumors are discovered before mastectomy, tumors can be removed more precisely. This requires routine breast cancer screening before every mastectomy. Nevertheless, routine screening in young individuals might be unnecessary since their overall low risk of breast cancer [54,55]. Screening could then lead to false-positive results, causing stress and unnecessary radiation, costs, and therapy [54]. Moreover, mammography can be stressful for FtM transsexuals since the test is not consistent with their male gender identity [56].

As it is unclear whether FtM transsexuals without mastectomy have a higher or lower risk of developing breast cancer compared to natal women, it is unclear at what age screening for breast cancer would be effective. Two studies recommend population screening of FtM transsexuals according to current guidelines of natal women [[55], [56], [57], [58]]. One study additionally recommends shared decision-making in order that FtM transsexuals can decide together with their physicians if they need screening, after having received information about the harms and benefits of screening [59]. Another study additionally recommends screening transsexuals with a history of five years of hormone therapy [60]. Both these recommendations can likely be used to determine if it is necessary to screen prior to mastectomy.

Including our own case, in nine cases the breast tumor was incidentally found by routine histological examination after mastectomy (Table 1). Routine histological examination of the breast tissue after every mastectomy is recommended in the literature [18,61]. This is recommended for reduction mammoplasty as well [62]. However, both these conclusions are based on only a few cases. It is difficult to make evidence-based recommendations based on the current literature. Routine histopathological screening after mastectomy could have relevant clinical consequences for at least the individuals who are at increased risk of developing breast cancer. Factors such as increasing age or positive family history could be taken into consideration. National guidelines can be used to assesses which individuals are at increased risk [55,63].

Prior to testosterone therapy for masculinization, our subject used anabolic-androgenic steroids, which are steroids that include synthetic substances that have similar effects as testosterone. We found no literature describing an association between the use of anabolic-androgenic steroids and an elevated risk of breast cancer development.

## Conclusion

Including our own case, we described 23 cases of FtM transsexuals developing breast cancer after testosterone therapy. Cases such as these make physicians aware of the possibility of breast cancer development in this population. Breast cancer screening of FtM transsexuals prior to mastectomy and histological examination of the mammalian tissue after mastectomy could be considered. In consultation with every individual FtM transsexual, physicians can decide if screening before and after mastectomy is necessary.

## Disclosure statement

The authors have no other financial or personal relationships relevant to this study to disclose. No funding was received for this article. There are no conflicts of interest to disclose.

## Funding

There was no funding for this study.

## Ethical approval

The patient gave verbal informed consent for this study. Formal consent is not required for the literature study.

## Declaration of competing interest

The authors declare that they have no conflict of interest.

## Figures and Tables

**Figure:**
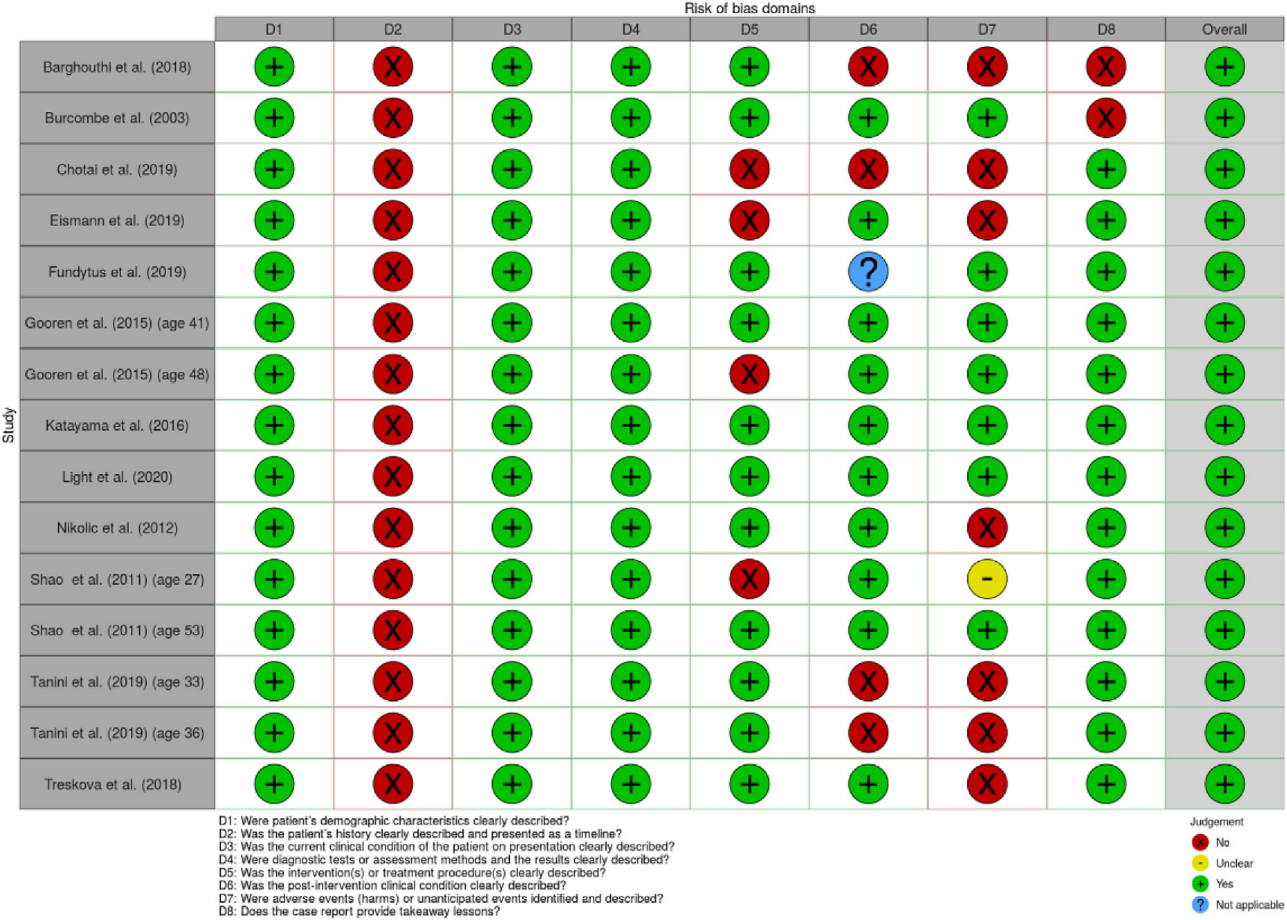


**Figure:**
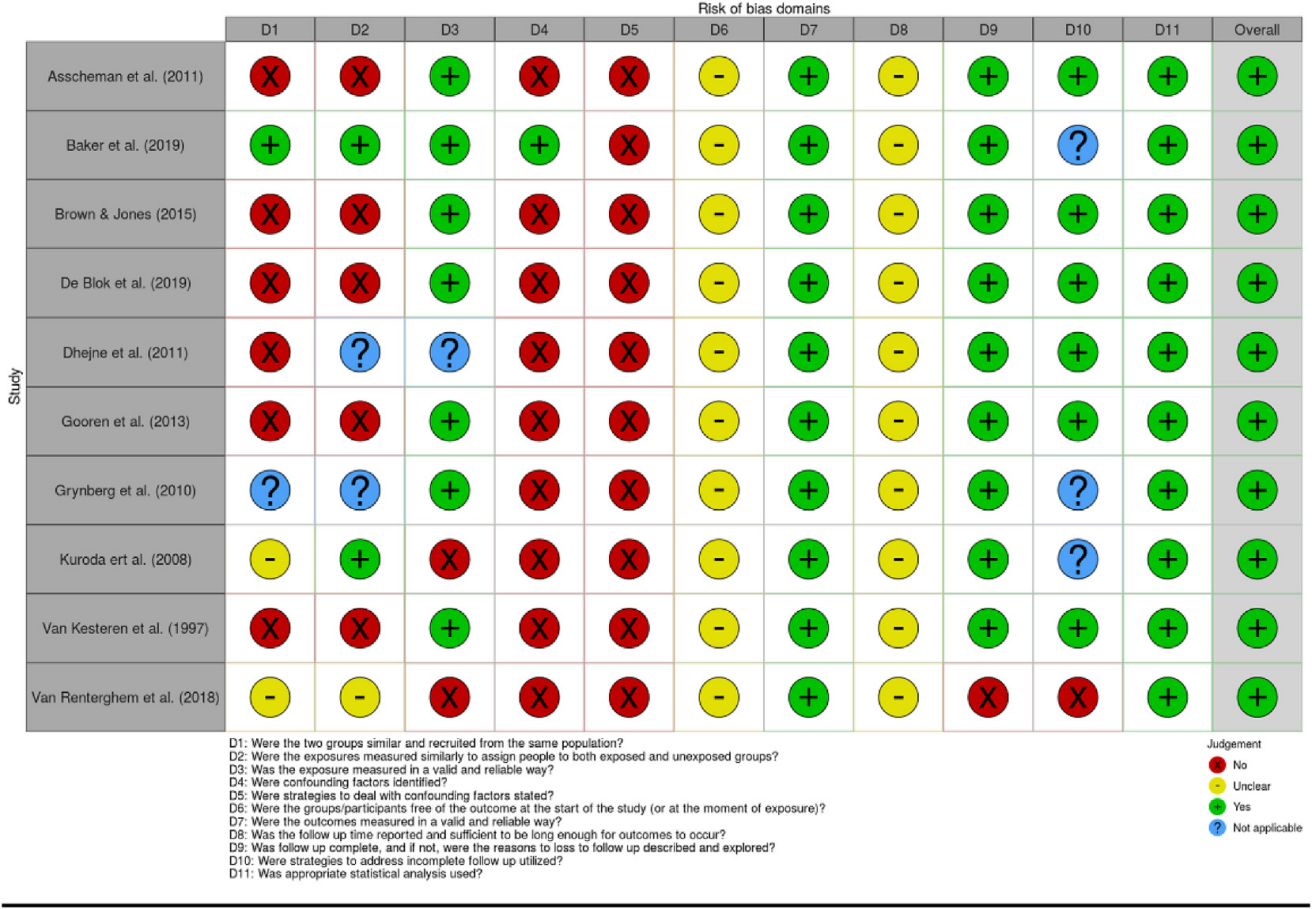


## Appendix 1 Search

Pubmed/MEDLINE search:

(Transgender Persons [Mesh] OR transsexualism [Mesh] OR transgender∗ [tiab] OR transsexual∗[tiab] OR intersex∗[tiab] OR female-to-male [tiab] OR FtM transsexual [tiab])

AND

(Breast Neoplasm[Mesh] OR breast cancer [tiab] OR breast malignancy [tiab] OR breast tumor∗ [tiab] OR breast carcinoma [tiab] OR mamma carcinoma [tiab] OR mammary neoplasm [tiab] OR mammary cancer [tiab])

AND

English[la]

NOT

(Clinical Conference[pt] OR Consensus Development Conference[pt] OR Consensus Development Conference, NIH[pt] OR Meeting Abstract[pt])

Ovid/EMBASE search:

1. exp transgender/
2. exp transsexualism/
3. exp intersex/
4. 1 or 2 or 3
5. (transgender∗ or transsexual∗ or intersex∗ or female-to-male or FtM transsexual).ti,ab,kw.
6. 4 or 5
7. exp breast tumor/
8. (breast cancer or breast malignancy or breast tumor∗ or breast carcinoma or mamma carcinoma or mammary neoplasm or mammary cancer).ti,ab,kw.
9. 7 or 8
10. 6 and 9
11. limit 10 to conference abstract status
12. 10 not 11
13. limit 12 to english language

## Appendix 2 Risk of bias assessment of case reports and cohort studies

Since we predetermined not to exclude studies based on their quality, we answered “yes” for the ‘overall judgment’ of each study.

Risk of bias for every individual case report:

Risk of bias for every individual cohort study:
